# Long-term preservation of bacteria in washed sterile sand: A 14-year study

**DOI:** 10.1371/journal.pone.0340501

**Published:** 2026-01-07

**Authors:** Amina Manni, El-Houcine Ait-Ouakrim, Bouchra Belkadi, Abdelkarim Filali-Maltouf

**Affiliations:** 1 Laboratory of Microbiology and Molecular Biology, Faculty of Sciences, Mohammed V University, Rabat, Morocco; 2 Laboratory of Epidemic Diseases, Department of Medical Bacteriology, National Institute of Hygiene, Rabat, Morocco; DIU: Dhaka International University, BANGLADESH

## Abstract

**Background:**

Cryopreservation and lyophilization are standard methods for preserving microbial cultures, but they are expensive and labor-intensive, creating a barrier for resource-constrained laboratories. This study evaluated the efficacy of a simple and inexpensive alternative: long-term storage in washed and sterilized sand.

**Methods:**

Fourteen bacterial strains from ten genera were preserved in sealed tubes containing washed and sterilized sand at room temperature. Viability, assessed by colony-forming units (CFU/g), was quantified at inoculation and after 7 and 14 years. Phenotypic stability was evaluated by comparing the pre- and post-storage profiles for enzyme activity and stress tolerance.

**Results:**

After seven years, all 14 strains remained culturable with preserved phenotypic features. After 14 years, 13 strains (93%) were viable. The highest recovery rates were observed in spore-forming Gram-positive bacteria (e.g., *Bacillus sp*.), indicating taxon-specific survival. Notably, the Gram-negative strain *Enterobacter hormaechei* remained stable with only a approximate 1.58 log₁₀ reduction over 14 years (half-life = 2.67 years). All resuscitated strains maintained their pre-storage characteristics. The distinct survival patterns for each species indicate that contamination was not the primary driver of the outcomes.

**Conclusion:**

Washed and sterile sand offers a practical, low-energy matrix for long-term preservation of a diverse range of bacteria, effectively maintaining cultivability and key phenotypic attributes for over a decade. This approach is an economical preservation strategy for resource-limited laboratories and field collections.

## Introduction

Biological research and biotechnological applications require genetically and phenotypically stable microbial cultures to be maintained [1,2]. Globally, culture collections primarily rely on cryopreservation and lyophilization, which are effective but require substantial infrastructure and continuous energy, making them challenging for laboratories in low-resource settings. It is particularly noticeable in areas of high biodiversity, where unique microbial isolates tend to disappear because of a lack of proximate methods of preservation [3,4]. Consequently, there is a growing interest in developing simple, affordable, and energy-independent preservation methods. Many microorganisms naturally withstand long periods of desiccation and nutrient scarcity by entering a state of metabolic arrest or sporulation [5]. Based on these natural resilience mechanisms, we suggest that a sterile inorganic matrix may provide a stable environment for long-term preservation. In particular, sand is a promising candidate [6]. When washed, it creates a nutrient-poor and inert environment that likely promotes microbial dormancy [7]. Although silica gel and paper have been explored for storage [8,9], comprehensive long-term data spanning decades are limited.

We selected a diverse panel of bacteria to rigorously assess the broad applicability of sand as a preservation matrix in various conditions. This included spore-forming Gram-positive bacteria, which are expected to survive well due to the well-known resilience of endospores [15,21], and more sensitive non-spore-forming Gram-positive and Gram-negative strains, such as *Pseudomonas*, *Acinetobacter*, and *Enterobacter hormaechei*. The inclusion of these sensitive strains provided a stringent test of the matrix’s efficacy, as their long-term survival is contingent upon less robust mechanisms such as metabolic dormancy and the accumulation of protective solutes [5,7,16–18]. This 14-year study was therefore conducted to examine the use of washed sterile sand as a preservation matrix, evaluating the long-term cultivability of 14 different bacterial strains and the stability of their key phenotypic properties.

## Materials and methods

### Ethics statement

Field sampling consisted solely of small volumes of inert sand collected in the northeastern desert of Morocco from a public, non-protected site. No plants, animals, or living microbial communities were collected at the field site, and no human participants were involved. According to the policies of the relevant local municipal authority, no permit is required for the small-volume collection of inert sand on public, non-protected land for academic research. Samples were transported in sealed containers to the Laboratory of Microbiology and Molecular Biology (Faculty of Sciences, Mohammed V University, Rabat), and all laboratory work complied with institutional biosafety guidelines.

### Bacterial strains and growth conditions

Fourteen bacterial strains chosen randomly from our laboratory collection, previously studied [10], were used in this study. The strains represented 10 genera: *Acinetobacter, Agrobacterium, Bacillus, Enterobacter, Erwinia, Ornithinibacillus, Paenibacillus, Pseudomonas, Stenotrophomonas and* S*taphylococcus*. The studied strains (Table 1) were cultivated in nutrient broth at 28°C with aeration until reaching the stationary phase (~10⁹ CFU/mL).

**Table 1:** The strains’ GenBank accession numbers.

Strain	GenBank accession number
*Acinetobacter johnsonii*	KX013413.1
*Agrobacterium tumefaciens*	KX013415.1
*Bacillus sp*	KX013406.1
*Pseudomonas oleovorans*	KX013430.1
*Enterobacter hormaechei*	KX013419.1
*Enterobacter sp*	KX013418.1
*Erwinia sp*	KX013420.1
*Ornithinibacillus scapharcae*	KX013424.1
*Paenibacillus lautus*	KX013425.1
*Pseudomonas knackmussii*	KX013427.1
*Pseudomonas sp*	KX013431.1
*Staphylococcus epidermidis*	KX013436.1
*Staphylococcus sp*	KX013438.1
*Stenotrophomonas rhizophila*	KX013439.1

### Sand preparation and inoculation

**Fig 1:**
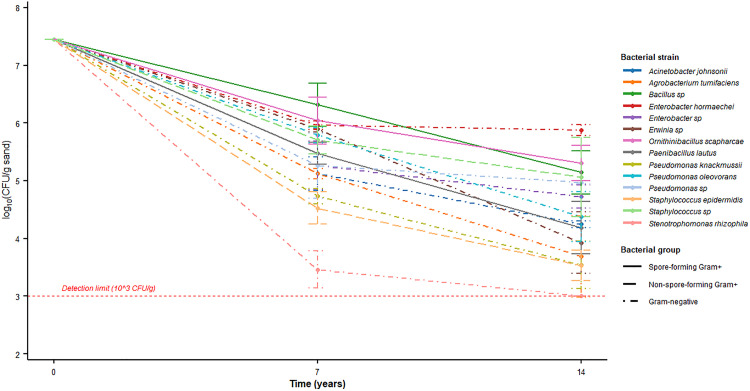
Bacterial viability during sand storage. CFU per gram of sand for 14 strains at inoculation (0 years), 7 years, and 14 years plotted on a log₁₀ scale. Points are means of *n* = 3 technical replicates per strain and time point (except *Enterobacter hormaechei*, 2021, *n* = 2); error bars represent standard deviations. Solid lines indicate spore-forming Gram-positive strains, dashed lines non-spore-forming Gram-positive strains, and dotted lines Gram-negative strains. The dashed red line marks the detection limit (~10^3^ CFU g ⁻^1^); values below the limit were plotted at the limit. Underlying data are provided at Figshare (https://doi.org/10.6084/m9.figshare.30178384).

The sand collected from the desert was carefully rinsed with distilled water until the effluent was clear to remove soluble nutrients. The material was then dried and sterilized by autoclaving at 121°C for one hour. After autoclaving, representative 1g sand subsamples were suspended in sterile buffer and plated (100 µL) on nutrient agar and incubated at 28 °C for 48 hours. The physicochemical parameters represent 95.4% of sand, a pH of 8.5, and 0.18% of organic content. A 100 μL dose of bacterial culture of each of the strains was injected into a tube (3.5 g sterile sand). Control tubes containing 3.5 g of sterile sand inoculated with 100 μL of sterile nutrient broth were pre-prepared and kept under the same conditions (Fig 1). All of the tubes were put under the seal, vortexed and stored under dark conditions at room temperature.

### Viability assessment

The sampling intervals of 7 and 14 years were selected to assess long-term survival throughout a timeframe pertinent to microbial preservation, while reducing the risk of contamination from the frequent unsealing of the tubes. Viability was evaluated at inoculation (year 0, 2007) and subsequently at 7 (2014) and 14 years (2021). Each strain was treated in triplicate at each time point. Aseptically, 0.1 g of sand was suspended in 1 mL of sterile physiological saline (0.9% NaCl) and vortexed for 1 min, serially diluted, and spread over nutrient agar plates. The plates were incubated at 28°C for 24–48 hours. Colony-forming units (CFU) were enumerated and expressed as CFU per gram of sand. The sterility of all control tubes was meticulously evaluated at each time interval. All manipulations were performed aseptically using sterile disposables. At each time point, in parallel with inoculated tubes, we processed (i) blank control tubes (sterile sand + sterile nutrient broth, no inoculum) and (ii) handling blanks (sterile buffer only); aliquots from both controls were plated under the same conditions.

### Phenotypic characterization

All strains were tested by qualitative assays of hydrolytic enzymes (cellulase, lipase, protease and amylase) and NaCl tolerance (1–13%) and temperature tolerance (25–55°C) by conventional plate assays prior to storage and after revival [11–14].

### Statistical analysis

Analyses were performed in R (v4.3.0). CFU g ⁻^1^ values were log₁₀-transformed for summaries and tests. For each strain and time point, three technical replicates were measured; data are presented as mean ± SD of log₁₀ (CFU g ⁻^1^). Paired, two-sided Wilcoxon signed-rank tests compared strain-level viabilities between 7 and 14 years (n = 13 pairs, α = 0.05); we report the Hodges–Lehmann median paired difference (14–7 years) with 95% CIs. Half-lives were estimated from log-linear models of log₁₀ (CFU g ⁻^1^) at 0, 7, and 14 years; t₁⁄₂ = log₁₀ (0.5)/slope (negative slopes yield positive half-lives). Values below the detection limit (~10^3^ CFU g ⁻^1^) were treated as left-censored at the limit for analysis.

## Results

### Viability after long-term storage

After seven years of preservation all the fourteen bacterial strains were recovered successfully. The viability of culturing (Fig 1) was subsequently determined to be 93%, with 13 out of the 14 strains being viable after 14 years. Blank control tubes (sterile sand and sterile nutrient broth, devoid of inoculum) maintained and analyzed alongside inoculated tubes exhibited 0 CFU at both the 7 and 14-year intervals. this confirms the long-term sterility of the preservation matrix. Furthermore, the consistent absence of growth in the handling blanks verified the integrity of our aseptic technique during sampling. Control tubes exhibiting contamination (one isolated case at 7 years and another at 14 years) were eliminated from the analysis, underscoring the rigor of our sterility monitoring.

Viability declined significantly between 7 and 14 years (paired Wilcoxon signed-rank test, V = 0, p = 0.000244). The Hodges–Lehmann median paired change from 7 to 14 years was −0.97 log₁₀ CFU g ⁻^1^ (95% CI −1.25 to −0.66; n = 13 strains). *Enterobacter hormaechei* declined only 0.08 log₁₀ units between 7 and 14 years (t₁⁄₂ ≈ 2.67 years), while spore-formers (*Bacillus sp., Ornithinibacillus scapharcae*) showed estimated half-lives of 1.82 and 1.96 years, respectively. In contrast, *Stenotrophomonas rhizophila* reduced to the detection limit (10^3^ CFU/g) after 14 years, with counts regarded as censored in subsequent analyses. Sterility of control tubes was strictly checked over the 14-year storage time. It was determined that one control tube was contaminated at the 7-year mark and was subsequently eliminated. At the end of the analysis at 14 years, another control tube was contaminated and was not included in the final results. The pattern of species-specific survivability in the experimental replicates (with each strain having its own characteristic predictable decay kinetics) do not conform to random contamination, and indicate strongly that the recovered cells were of the original inoculum. The probability of survival was very strain-dependent (Table 2). The gram-positive organisms, sporulating (e.g., *Bacillus sp*., *Ornithinibacillus scapharcae*), exhibited the greatest vitality, with final concentrations exceeding 10⁵ CFU/g. Among Gram-negative organisms, *Enterobacter hormaechei* exhibited remarkable stability, decreasing by merely ~1.58 log_10_ over the span of 14 years (half-life ~2.67 years). Conversely, *Stenotrophomonas rhizophila* exhibited complete loss of cultivability by the 14-year mark.

**Table 2:** Survival metrics and phenotypic stability of bacterial strains after 7 and 14 years of storage. Log₁₀ reduction in viable counts relative to the initial inoculum after 7 and 14 years of storage in sterile sand, and estimated half-life for each strain assuming exponential (log-linear) decay of CFU g ⁻^1^ over time. Values are mean ± SD of log₁₀ (CFU g ⁻^1^) based on three technical replicates per time point (except *Enterobacter hormaechei* at 14 years, *n* = 2). Phenotypic stability indicates that the original enzymatic activity and stress-tolerance profile were maintained after storage. N/A, not estimable due to counts at or below the detection limit at 14 years.

Strain	Gram	Log₁₀ Reduction (mean ± SD)		Estimated Half-life (years)	Phenotypic stability	
		7 years	14 years		7 years	14 years
*Acinetobacter johnsonii*	–	2.33 ± 0.29	3.21 ± 0.06	1.31	Yes	Yes
*Agrobacterium tumefaciens*	–	2.30 ± 0.05	3.53 ± 0.66	1.19	Yes	Yes
*Bacillus sp.*	+	1.20 ± 0.33	2.31 ± 0.37	1.82	Yes	Yes
*Enterobacter hormaechei*	–	1.50 ± 0.07	1.58 ± 0.07	2.67	Yes	Yes
*Enterobacter sp.*	–	2.00 ± 0.15	2.66 ± 0.13	1.58	Yes	Yes
*Erwinia sp.*	–	1.54 ± 0.02	3.53 ± 0.53	1.19	Yes	Yes
*Ornithinibacillus scapharcae*	+	1.24 ± 0.17	2.15 ± 0.31	1.96	Yes	Yes
*Pseudomonas knackmussii*	–	2.76 ± 0.08	3.91 ± 0.41	1.08	Yes	Yes
*Paenibacillus lautus*	+	1.91 ± 0.11	3.27 ± 0.45	1.29	Yes	Yes
*Pseudomonas oleovorans*	–	1.71 ± 0.06	3.08 ± 0.43	1.37	Yes	Yes
*Pseudomonas sp.*	–	2.28 ± 0.49	2.48 ± 0.03	1.70	Yes	Yes
*Staphylococcus epidermidis*	+	2.99 ± 0.22	3.92 ± 0.27	1.07	Yes	Yes
*Staphylococcus sp.*	+	1.82 ± 0.15	2.39 ± 0.69	1.77	Yes	Yes
*Stenotrophomonas rhizophila*	–	4.06 ± 0.25	N/A	N/A	Yes	No

### Phenotypic stability

All 13 strains recovered after 14 years preserved their pre-storage phenotypic characteristics (S2 and S3 Figs). The patterns of hydrolytic enzyme synthesis and resistance to NaCl and thermal stress were essentially consistent.

## Discussion

Despite the differences in survival rates of the strains, this study demonstrates that bacteria can remain viable and phenotypically stable for over ten years at ambient temperature in sterile sand. All isolates were recoverable after seven years, and thirteen out of fourteen were still cultivable after fourteen years. These results demonstrate that sand is a convenient preservation matrix, but they also suggest that the physiology and taxonomic group of the organisms play a prominent role in the results. While storing bacteria in inert matrices is not a novel [8,9], our study provides unprecedented quantitative evidence of its effectiveness over a decade. More importantly, by using washed sand, we demonstrate that long-term survival can be achieved through a passive, nutrient-independent method of preservation, which is different in nature to methods that rely on lyophilisation or cryoprotectants. This implies that the sand matrix in itself provides a stabilising environment that mitigates the physico-chemical stresses linked with long-term preservation, and does not act as a simple container. The high performance of the Gram-positive spore-forming bacteria is consistent with their ability to develop latent states.

The sterile sand matrix preserves low water activity and little nutrients, creating conditions that intrinsically stabilize endospores. The enhanced long-term survival of *Bacillus* and *Ornithinibacillus* aligns with the known resistance mechanisms of spores. These factors encompass core dehydration, the presence of calcium-dipicolinate, DNA protection by small acid-soluble proteins (SASPs), and a protective cortex and coat that jointly reduce macromolecular damage during extended desiccation [15,20]. The inherent longevity is additionally governed by intricate germination pathways that remain dormant in the steady sand environment [21]. The success of this strategy in specific strains, independent of their short-term stress tolerance profiles, explains why survival over a decade was not predictable from standard physiological assays and was not universal among all Gram-negative taxa. Non-spore-forming bacteria, such as the exceptionally hardy *Enterobacter hormaechei* we studied, likely survive by various adaptive methods. This involves entering dormant or viable-but-non-culturable (VBNC) states to reduce metabolism, accumulating compatible solutes like trehalose to stabilize cellular structures, remodeling membrane lipids to maintain integrity at low water activity, and activating repair systems upon rehydration [5,7,16–18].

The *Bacillus* species and *Ornithinibacillus scapharcae* had estimated half-lives of approximately 2 years, hence had the highest final CFU counts. This supports the known role of spores in resisting nutrient limitations and long-term desiccation [15]. Although *S. epidermidis* declined more rapidly, other *Staphylococcus* species also survived at relatively high levels, suggesting that persistence depends on factors beyond sporulation. The most interesting finding was that Gram-negative bacteria had a remarkable exception of *Enterobacter hormaechei,* which had remarkable stability. Its longevity (a 1.6-log decrease over 14 years) suggests that some non-spore formers may be using other survival strategies, and these could include biofilm formation, a buildup of protective solutes such as trehalose, or other dormancy strategies [16]. Showing that sand storage can be useful not only for spore-forming organisms but also for other organisms makes this study particularly significant. The idea that Gram-negative bacteria are inherently unsuitable for long-term, non-cryogenic storage is challenged by the resilience of *E. hormaechei*. This makes this strain, and perhaps others with similar strategies, valuable models for studying the molecular and genetic basis of persistent dormancy in non-sporulating bacteria. Although dry preservation utilizing inert matrix such as silica-based and other inorganic systems has been previously established, these investigations generally encompassed shorter durations and limited taxonomic diversity [8,9]. Our 14-year dataset significantly extends this evidence. Our findings confirm the exceptional survival of spore-forming Gram-positive bacteria, which aligns with known spore resistance mechanisms [15,20]. More importantly, we provide robust evidence of measurable persistence in non-spore-forming Gram-negative strains, most notably *Enterobacter hormaechei.* This supports growing evidence that such bacteria can endure long-term desiccation through mechanisms like dormancy, trehalose accumulation, and cellular repair programs [5,7,16–18]. Consequently, our work demonstrates that decade-scale survival in a sterile sand matrix is not an exclusive trait of spore-formers. Interestingly, long-term survival was not predicted by short-term stress tolerance, such as resistance to heat or salt (S3 Fig). While *E. hormaechei*, with modest stress tolerance, remained robust, *Stenotrophomonas rhizophila*, which thrived in 4% NaCl, vanished entirely. This pattern suggests that the mechanisms enabling long-term metabolic arrest in sand are different from those supporting survival during daily or weekly stress exposure [5,7,17,18]. It is important to note that we only analysed culturable cells. According to reports on other environmental bacteria, some strains may have persisted in a viable but non-culturable (VBNC) state [17,18]. Thus, our data provide a solid indicator of culturable survival, but also form a baseline of future studies to understand the full scope of the survival, including entering the VBNC state (via viability staining or molecular activity assays). Although we quantified cultivability and phenotypic profiles, we did not measure growth kinetics; future work will quantify recovery kinetics (lag time) and maximum specific growth rate (μmax) in standardized broth assays and colony expansion on agar for pre-storage and revived isolates to assess physiological state and resuscitation potential. Maintaining phenotypic characteristics was an outstanding result. All the remaining strains had identical tolerances to temperatures and salt concentrations, and equal enzyme activity (cellulase, lipase, amylase, and protease). This means that both functional stability and viability are maintained in sand storage. Preservation of the hydrolytic activity of enzymes has possible biotechnological uses as well as ecological importance, such as in desert soil survival [19]. Overall, our results enhance understanding of what constitutes a preservation environment. They demonstrate that a simple, inert, nutrient-free matrix can induce a state of metabolic pause that maintains both viability and function, eliminating the need for additional protectants. Our data confirmed the preservation of cultivability and essential phenotypic traits; however, a thorough assessment of growth kinetics, encompassing lag phase, doubling time, and maximal specific growth rate, warrants further investigation. This data would offer enhanced understanding of the physiological condition and recovery capabilities of cells reanimated from prolonged storage. The observed stability of the hydrolytic and tolerance profiles indicates that the fundamental metabolic capabilities necessary for growth and function were preserved. Despite the stability of phenotypic characteristics, forthcoming whole-genome sequencing of pre-storage and revived isolates will assess genomic alterations and verify strain authenticity. Additionally, we will assess potential horizontal gene transfer by pan-genome comparison and plasmid/mobile-element profiling between pre-storage and revived isolates.

The success of this approach depends on mimicking the sterile, stable conditions of a natural habitat rather than reproducing growth conditions in a laboratory. Practically, storage of sand can be considered as a complement to either cryopreservation or lyophilisation rather than as an alternative. It is particularly applicable in resource-constrained regions since it is simple, economical and energy-independent. The primary weaknesses include the variability of strain and partial universality. Nevertheless, as demonstrated here, it has the promise of maintaining a wide set of taxa and their functional characteristics over a period of over a decade, which makes it a promising backup or field-deployable approach towards the conservation of microbial resources in biodiverse, resource-limited environments [20,21]. Importantly, the bacterial collection remains conserved in sterile sand in our laboratory under the same conditions, and we will reassess its viability after 21 years in total to further re-evaluate this preservation method.

## Conclusion

This 14-year research presents good evidence that washed and sterile sand is a viable method of preserving bacteria, their viability, and the functional property that is cost-effective and reliable. Its total autonomy from energy sources or complex infrastructure makes it a crucial instrument for preserving microbial genetic resources in field stations and resource-constrained laboratories worldwide.

## Supporting information

S1 Fig Storage of bacterial strains in washed sterile sand. (A) Overview of glass vials containing 3.5 g of washed, autoclaved desert sand inoculated with bacterial cultures and sealed with screw caps, arranged in a polystyrene rack. (B) Close-up view showing the sand-filled vials used as the long-term preservation matrix, stored in the dark at room temperature for up to 14 years.
(TIF)

S2 Fig Strains’ preserved enzymatic activities. The heatmap shows preserved extracellular enzymatic activities of bacterial strains after 14 years of storage in washed sterile sand. Each row corresponds to a strain and each column to one hydrolytic enzyme (cellulase, lipase, protease and amylase). Dark cells indicate detectable activity (present), and light cells indicate no detectable activity (absent) under the tested conditions.
(TIF)

S3 Fig Correlation between osmotic characteristics and strain viability (A: tolerance to salinity, B: tolerance to temperature). (A) Survival in 2021 (expressed as percentage of initial CFU in 2014, log scale) as a function of maximum NaCl tolerance (%) for each strain. (B) Survival in 2021 as a function of the midpoint of the temperature tolerance range (°C). Each point represents one strain. Pearson’s and Spearman’s correlation analyses showed no clear association between either NaCl tolerance or temperature tolerance and long-term survival.
(TIF)
